# Weighted assignment fusion algorithm of evidence conflict based on Euclidean distance and weighting strategy, and application in the wind turbine system

**DOI:** 10.1371/journal.pone.0262883

**Published:** 2022-01-24

**Authors:** Liming Gou, Jian Zhang, Naiwen Li, Zongshui Wang, Jindong Chen, Lin Qi

**Affiliations:** 1 School of Business Administration, Liaoning Technical University, Huludao, Liaoning, China; 2 School of Economics and Management, Beijing Information Science & Technology University, Beijing, China; 3 Laboratory of Big Data Decision making for Green Development, Beijing, China; 4 Beijing International Science and Technology Cooperation Base of Intelligent Decision and Big Data Application, Beijing, China; University of Defence in Belgrade, SERBIA

## Abstract

In the process of intelligent system operation fault diagnosis and decision making, the multi-source, heterogeneous, complex, and fuzzy characteristics of information make the conflict, uncertainty, and validity problems appear in the process of information fusion, which has not been solved. In this study, we analyze the credibility and variation of conflict among evidence from the perspective of conflict credibility weight and propose an improved model of multi-source information fusion based on Dempster-Shafer theory (DST). From the perspectives of the weighting strategy and Euclidean distance strategy, we process the basic probability assignment (BPA) of evidence and assign the credible weight of conflict between evidence to achieve the extraction of credible conflicts and the adoption of credible conflicts in the process of evidence fusion. The improved algorithm weakens the problem of uncertainty and ambiguity caused by conflicts in the information fusion process, and reduces the impact of information complexity on analysis results. And it carries a practical application out with the fault diagnosis of wind turbine system to analyze the operation status of wind turbines in a wind farm to verify the effectiveness of the proposed algorithm. The result shows that under the conditions of improved distance metric evidence discrepancy and credible conflict quantification, the algorithm better shows the conflict and correlation among the evidence. It improves the accuracy of system operation reliability analysis, improves the utilization rate of wind energy resources, and has practical implication value.

## Introduction

Information fusion technology has solved many troubles [1–7] in the military, engineering, and environment since it developed in the 1970s [8]. The application have expanded to much more areas, such as extra energy, new materials, manufacturing, medicine, agriculture, transportation, and economy [9–15]. The utilization of information fusion technology enhances the system fault tolerance, self-adaptability, and reduces inference fuzziness. It meets the requirement of traditional algorithms for a priori probability and provides a basis for event decision-making. Its typical features make it widely used in fault diagnosis, anomaly detection, reliability, inference, prognosis, and early prediction [16–19].

In the information explosion era, information presents a massive, multi-source, heterogeneous, multi-dimensional, complex, and fuzzy feature. It has developed rapidly emerging information technology. The development of intelligence has significantly increased the complexity of the various levels of the system, which makes the system faces reliable operation challenges [20, 21]. Under this condition, the Chinese government actively encourages researchers to organize fundamental research on the reliable operation of important equipment and components in key areas, including extra energy, energy conservation, emission reduction, and environmental protection. The data-driven multi-source information fusion technology has become one concern of system operation reliability research.

With extended the prior research, the Dempster-Shafer theory (DST) fusion algorithm has achieved better performance in comprehensive system state analysis and decision making. However, it has a strong subjective dependence [22] on basic probabilities assignment (BPA) and the independence of evidence, ​and the correlation relationship between evidence affects the fusion [23]. There are even troubles with distortion and disorder in the practical application process. Thus, based on previous studies, this study argues that quantifying the correlation between evidence and fairly assigning the fusion weights of evidence features is crucial to the fusion results. In response to these questions, researchers have studied the DST fusion algorithm from the perspectives of fusion framework, weight allocation, and method combination.

In terms of fusion frameworks, researchers have proposed different framework models, which improved the algorithm effectiveness. Brommer et al. [24] proposed a modular multi-sensor fusion framework, which is better efficient in dealing with delayed statistics collection, disordered updates, and monitoring the health of sensors themselves in complex systems. Xiao [25] discussed the modeling of uncertainty based on the framework of Triangular fuzzy numbers for fuzzy complex event processing systems in an uncertain environment, and proposed a fault-tolerant and reliable strategy for scheduling. Wang et al. [26] dealt with evidence conflicts in DST under the framework of fuzzy preference relationships, which improved the diagnostic accuracy of hybrid classifier integration. Prior research improved the idea and effectiveness of the integration to different degrees under the idea of modularity and different attention allocation.

To deal with the diversity, uncertainty, and conflict of information, researchers have proposed ideas of feature correlation, difference, different conflict values, and non-similarity measures. They improved and integrated algorithms [27–31] from mathematical perspectives, such as mean, combination, and entropy. Zhang et al. [32] proposed a method incorporating fuzzy object elements, Monte Carlo simulation, and DST, through weighted averaging and data deblurring rules, the result has clear analytical values to represent the final risk level. Xiao [33] combined the complex D-S theory and Quantum mechanics, to express and handle the uncertain information in the framework of the complex plane, and reduce the interference effects caused by uncertainty.

Wu et al. [34] proposed an improved evidence aggregation strategy combining the Dempster-Shafer rule and the weighted average rule. It overcomes the counterintuitive dilemma existing in the high conflict evidence combination by constructing the BPA under relevance metric. Jiang et al. [35] used evidence theory to model uncertainty, adopted a weighted average combination method to merge BPAs. Finally, it validated the method by motor empirical cases under the decision rules. Li et al. [36] proposed a weighted conflicting evidence combination method based on Hellinger distance and belief entropy., and uses distance to measure the conflict between evidence and applies belief entropy to quantify the uncertainty of basic belief assignments.

Under the Dempster-Shafer framework, Tang et al. [37] proposed a weighted belief entropy which is based on Dunn’s entropy, to quantify the uncertainty in uncertain information and reduce information loss during information processing. Ullah et al. [38] designed a data fusion scheme based on improved BPA belief entropy and quantified the uncertainty in information and transform conflicting data into decision results. The simulation result showed that the proposed scheme had stronger performance in terms of uncertainty, reason, and decision accuracy in an intelligent environment. Brumancia et al. [39] proposed an information fusion algorithm for decision making under different information conditions, which is based on D-S theory and adaptive neuro-fuzzy reasoning (DSANFI) system, it has widely used in robotics, statistics, control, and other fields.

From the researchers’ exploration, the information fusion algorithm based on Dempster-Shafer has always been a hot focus of research, which has a broad theoretical and practical value. In the current development process, the widespread application of intelligent systems increases the demand for system operation and maintenance. However, the existing algorithms [40–42] still have different degrees of information loss, fusion disorder, and low fusion accuracy in practical application, and the algorithms have the problem of universality [43].

Some studies [44–46] suggested that the main problem of the affected fusion results are the incomplete identification framework of evidence features, and the basic reliability probability of evidence is difficult to calculate completely and accurately, which lead to information loss and disorder. Therefore, in this research, the DST fusion model is promoted from the perspectives of the knowledge fusion framework, quantification of correlations, and extraction of credible conflicts to overcome the information loss problem.

## Propose a fusion framework

The multi-source information fusion problem in this paper refers to integrating multiple sources of information. Multiple sources are information originates from different means of monitoring the same part of the same thing. Therefore, in our proposed fusion framework, the multi-source information fusion problem [47] is summarized as a ternary problem, as shown in Eq (1).


$$Q=\{N_{i};<N_{i}>;D\}$$ (1)

Where, *N*_*i*_, *<N*_*i*_*>*, and *D* represent data, features, and decisions respectively.

**Fig 1:**
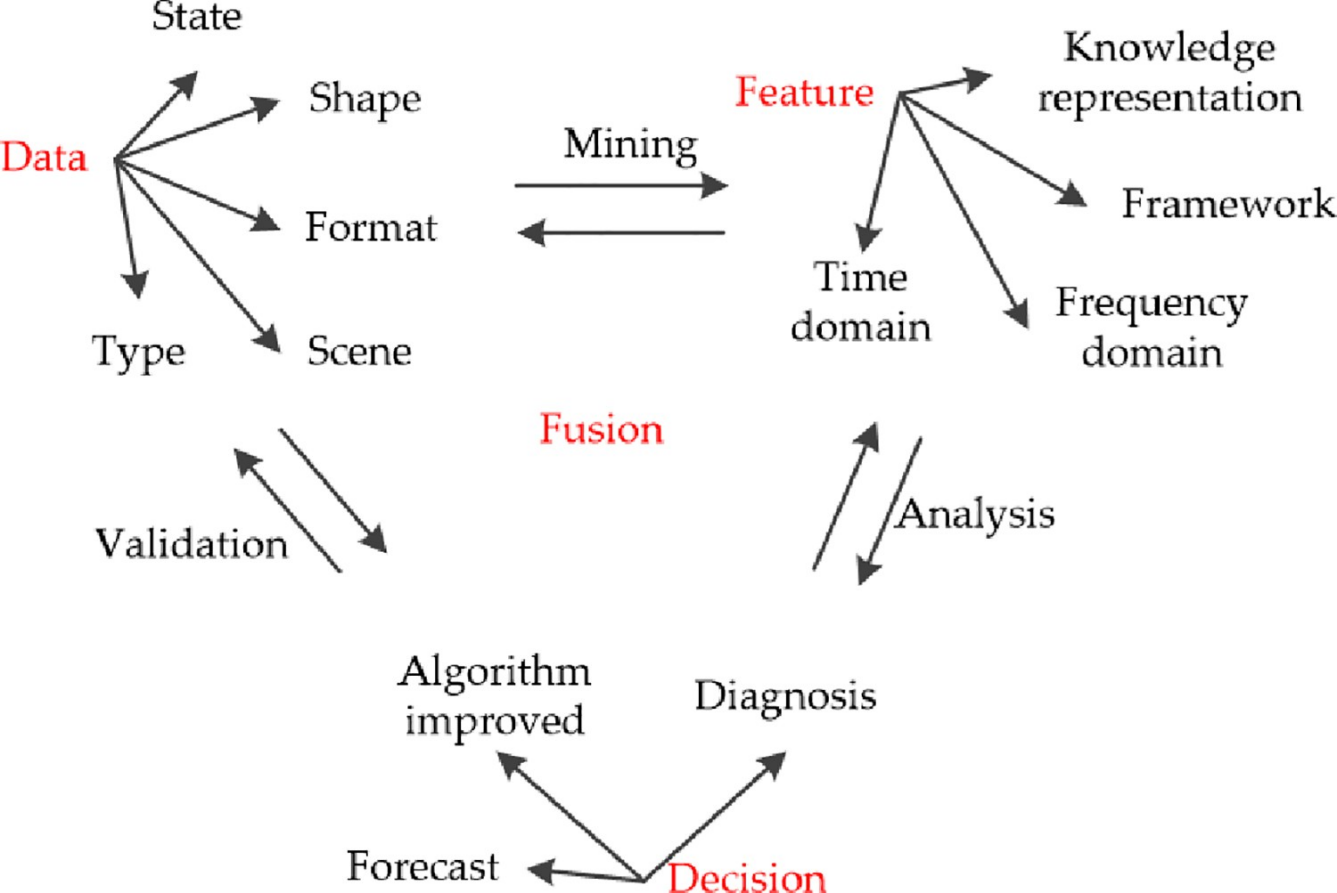
Ternary problems in multi-source information fusion.

The type, state, format, and scenario of data lead to its multi-source heterogeneity and complexity in the information management. Data set *N*_*i*_ contains an enormous amount of information, and the information is represented in the knowledge form, and the data feature set *<N*_*i*_*>* is constructed by mining the information of potential features’ data from the perspective of knowledge management. The knowledge is fused with algorithms to improve the recognition framework, and the accuracy and reliability of algorithms in the fusion process are improved to provide the foundation for management decisions. The relationship between data, features, and decisions is shown in Fig 1.

Data fusion is mainly reflected by the fusion of data features. It fuses the features exhibited by multiple homogeneous or heterogeneous data in the time or frequency domain which is beneficial to decision making. Considering the different data exhibits different features, assuming that *V*_*i*_ is different perspectives, then there is some correspondence between the whole process from the mapping of perspective space to data space and then to data feature space, as shown in Eq (2). When the data features or attributes cannot be directly fused, some kind of consistency processing needs to be performed before fusion.


$$\left(\begin{array}{c} V_{1}\\ V_{2}\\ \ldots \\ V_{i} \end{array})\ldots \Rightarrow \ldots (\begin{array}{c} {\vec{N}}_{1}\\ {\vec{N}}_{2}\\ \ldots \\ {\vec{N}}_{m} \end{array})\ldots \Rightarrow \ldots (\begin{array}{c} <{\vec{N}}_{1}>\\ <{\vec{N}}_{2}>\\ \ldots \\ <{\vec{N}}_{m}> \end{array}\right)$$ (2)

**Fig 2:**
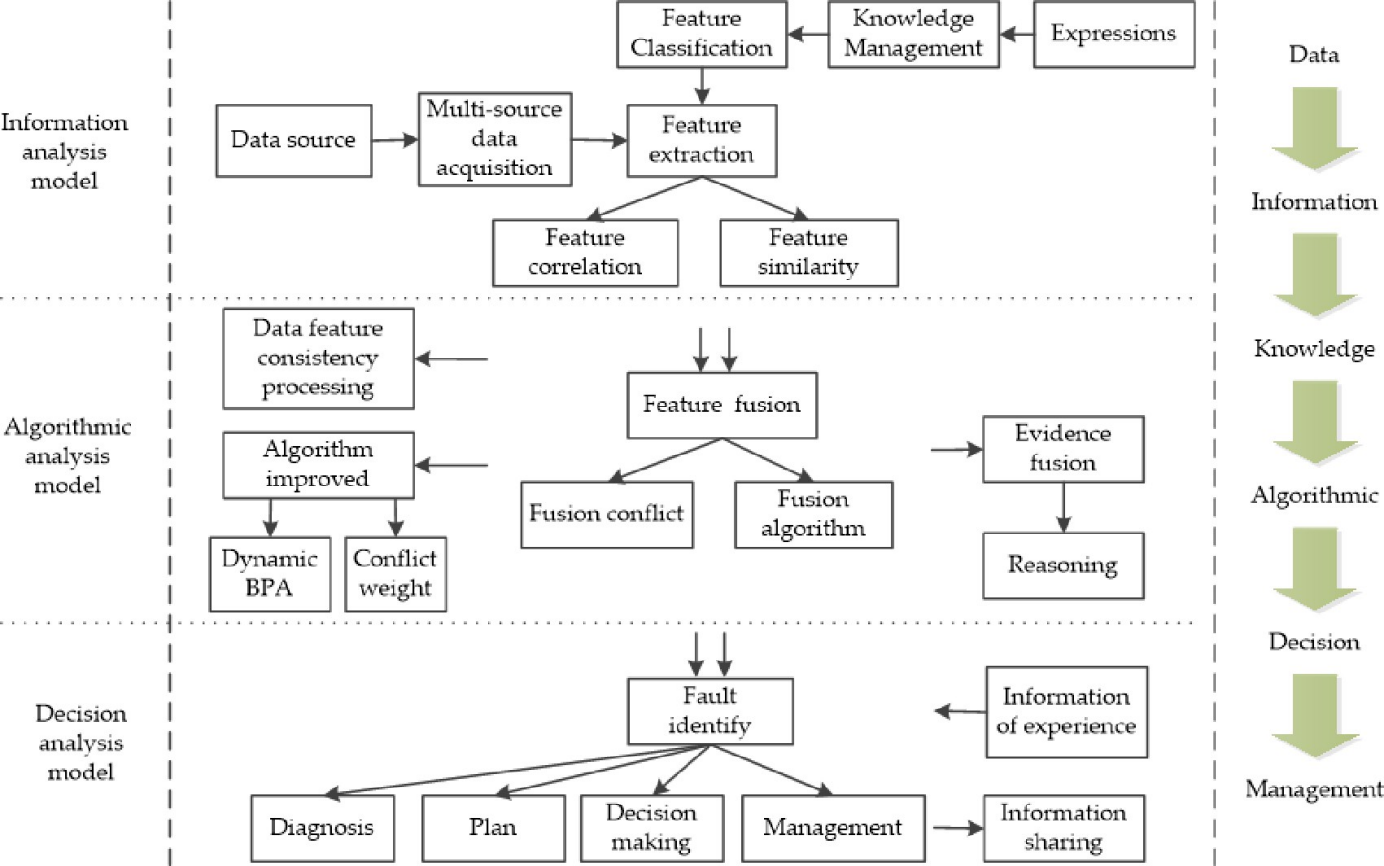
Fusion analysis framework.

It studies the multi-source information fusion analysis framework from three perspectives: information, algorithm, and decision making, and presents the problems of data ambiguity, conflicting evidence, and low fusion degree in the fusion process. It takes data represented as knowledge and classifies information features from different sources. Considering features similarity and conflict, data features should be quantified and changing rules should be found out, to weaken data ambiguity and keep the potential value of information [48]. Regarding the shortage of algorithms, it deals with the consistency of features and adopts methods of conflict weight assignment to reduce the impact of evidence association and evidence conflict on the fusion results. According to the feature performance, it can make a judgment on the system condition, to rationalize the system failure management in time and effectively reduce the loss. The fusion analysis framework is shown in Fig 2.

## Materials and methods

This study divides the algorithm into two stages, including the BPA calculation session and the fusion session. In parts “Improved algorithm under the weighting strategy” and “Improved algorithm under Euclidean distance weighting strategy”, improvements to the BPA calculation process are proposed, in part “Fusion algorithm under Improved Euclidean distance weighting strategy”, improvements of the fusion session is proposed. The fusion improvements are based on the BPA calculation.

### Improved algorithm under the weighting strategy

The feature information in different data sources of the same type has a certain similarity, and the feature information in different heterogeneous data sources also has a certain similarity. Studying the homogeneity and heterogeneity of data, it is necessary to analyze the data similarity when analyzing faults, to reduce the repetitive calculation work. Therefore, it defines a formal concept of data feature similarity, which is the degree of the similarity of the features in the information. As known that the data set is composed of multiple data, so it can be as a matrix.

Therefore, the features of the data can be denoted as $<E_{i}{\vec{N}}_{j}>$, then the similarity between the corresponding features of the two data sets is denoted as $Sim(<E_{i}{\vec{N}}_{j}>,<E_{k}{\vec{N}}_{j}>)$. According to the data time domain, the data is paired by pair, and the weight of the *qth* pair of data features is denoted as *w*_*q*_, then the similarity between the features of the two sets can be expressed by Eq (3).


$$Sim(<E_{i}{\vec{N}}_{j}>,<E_{k}{\vec{N}}_{j}>)=\sum_{k}w_{q}Sim_{k}(<E_{i}{\vec{N}}_{j}>,<E_{k}{\vec{N}}_{j}>)$$ (3)

Where, *N*_*j*_∈*N*, *i≠k*, *i*, *j*, *k*, *q* is not equal to 0. The weight *w*_*q*_ are assigned according to the importance of the features characterized by the data and need to satisfy *w*_1_+*w*_2_+…+*w*_*i*_ = 1.

There is a similarity between evidence *i* and *j*, so it introduces the similarity factor *Si*. The weighting strategy is used to quantify the similarity between evidence features, then the specific formula for quantifying the similarity between the two evidence is shown in Eq (4).


$$Si=<E_{i}{\vec{N}}_{j}>*<E_{k}{\vec{N}}_{j}>/\sqrt{<E_{i}{\vec{N}}_{j}{>}^{2}+<E_{k}{\vec{N}}_{j}{>}^{2}}$$ (4)

Let the similarity of the evidence be *Simz*, then the similarity of evidence *i* is shown in Eq (5).


$$Simz=\sum \left(<E_{i}{\vec{N}}_{j}>*<E_{k}{\vec{N}}_{j}>/\sqrt{<E_{i}{\vec{N}}_{j}{>}^{2}+<E_{k}{\vec{N}}_{j}{>}^{2}}\right)$$ (5)

Then, it gets a set of similarity sequences of length n*(*n*−*1*)/*2*, where *n*>*1*. Each group of evidence that forms a series of similarities with other evidence and the number of similarity data between evidence *i* and other evidence is (*n-1)*.

Therefore, the total similarity between evidence *i* and other evidence can be expressed by Eq (6).


$$Sim=\underset{n-1}{\underset{︸}{\sum \left(Simz_{ij}\right)}}$$ (6)

Where, *i* is the specified evidence, and when *i* is fixed without change, the dynamic value is taken for *j*, *i≠j*.

Then, the weights of the evidence are assigned as shown in Eq (7).


$$w_{i}=Sim_{i}/\sum Sim$$ (7)

When the similarity of data features is high, the weight of evidence is correspondingly high, shows that the supporting evidence for a certain type of event occurrence is high. And it can use more complete evidence data for two types of evidence factors that have high similarities. When the similarity is low, the weight declines, means that the perspective of making a judgment on event occurrence between data may be different, rather than the completely untrustworthy evidence. So it adopts multiple evidence factors to mine valuable conflicting information, to improve the accuracy of system fault diagnosis.

Once the similarity of the characteristics of the evidence is mastered, it can perform a new fusion of the evidence.

### Improved algorithm under Euclidean distance weighting strategy

#### Degree of evidence variation

In practice, there are conflicts among evidence [49]. Conflict is a kind of information related to the similarity of data features and is likely to have some value. The BPA of the evidence shows the credibility level of the evidence and reflects consistency in the assignment of the basic credibility probability of the evidence to the focal elements. Therefore, this study performs dynamic extraction of BPA, and based on this, adjusts the weight of evidence under conflict conditions, assigns conflict coefficients to different focal elements, reduces the weight of evidence with lower confidence in the fusion process, to improve the reliability of fusion results.

According to the relation between the variation in historical data features and the reliability of the system, it sets a reasonable threshold value. Dynamically monitor and extract the frequency of data features emerging in different threshold ranges to get the BPA of dynamic changes, as shown in Eq (8).


$$P({\vec{A}}_{i})=\frac{f_{i}}{\sum f_{i}}$$ (8)

The primary methods to measure the correlation between data include distance measure, Pearson relationship coefficient, cosine similarity, and deviation measure. Among them, the Pearson relationship coefficient is usually used to measure the inconsistency of data scale, when there is a subjective judgment standard inconsistency scenario. The cosine similarity coefficient is acting on data sparsity. The deviation is to reflect the difference between the basic credible probability distribution of focal elements and the average similarity value, but its use of the average similarity value weakens the measure of the true difference of data. Euclidean distance is a simple method to measure the distance between two points in the m-dimensional space and especially has a significant advantage with integrity data. Therefore, this paper adopts distance [50] to reflect the difference between two sets of data. Assuming that the difference between two pieces of evidence *i* and *j* is *d*_*ij*_, to ensure that the data is positive, Eq (9) can express the calculation of the difference between two pieces of evidence.


$$d_{ij}=\sqrt{{\sum_{B\cap C=A}\left(m_{1}({\vec{A}}_{i})‐m_{2}({\vec{A}}_{j})\right)}^{2}}$$ (9)

When the difference between two pieces of evidence is high, the similarity between the evidence is low and the conflict is high. When the difference is low, the similarity between the evidence is high and the conflict low.

The total number of data on the variation among the evidence is n*(*n*−*1*)/*2*, where, n > 1. It aggregates the differences between one evidence and the other to get *n* sets of variation data.

Then, Eq (10) can express the difference between evidence *i* and all others that affects the conclusion.


$$d(m_{i}({\vec{A}}_{i}))=\sum d_{ij},\;i、j=1,2,\ldots ,n.$$ (10)

Normalizing the difference between evidence *i* and others is the difference of evidence *i*, which can be expressed by Eq (11).


$$d(m_{i})=\frac{d(m_{i}({\vec{A}}_{i}))}{\sum_{i=1}^{n}d(m_{i}({\vec{A}}_{i}))}$$ (11)

Where, *n* denotes the number of evidence, and the credibility of evidence *i* is low when it conflicts with other evidence at a high level.

#### Credible weight of evidence

The confidence level of the evidence reflects the credibility level of the evidence, and the similarity of the focal elements reflects the similarity of the evidence, which reflects the consistency in the assignment of the basic credibility probability of the evidence to the focal elements. So this is an entry point for adjusting the weight under conflict conditions. Assigning the conflict coefficient *K* to different focal elements *A*_*i*_ reduces the weight of evidence with lower confidence in the fusion process, thus increasing the weight of evidence with high confidence and improving the reliability of fusion results.

Confidence is the support of data features to the event results, and it is the trustworthiness of the evidence information. The confidence function on the identification framework can be expressed by Eq (12).


$$Bel(\vec{A})=\sum_{B\subseteq A}m(\vec{B})$$ (12)

The equation shows that the confidence function is the sum of the probabilities of event support for all subsets of that event, and *B* is a subset of *A*. The confidence function has a certain influence on the reliable transmission of the system.

The likelihood function is the degree to which the evidence information does not negate the occurrence of an event, and it shows that the likelihood function is the sum of the probabilities that the intersection with that event is not empty. In the identification framework, it can express the likelihood function in Eq (13).


$$Pl\;(\vec{A})=\sum_{B\cap A\neq \phi }m(\vec{B})$$ (13)

The likelihood function contains both credible and implausible information, as shown in Eq (14). Therefore, the credibility of the evidence needs to be analyzed.


$$Pl(\vec{A})\to (Cre(m_{i}),\;nCre(m_{i}))$$ (14)

There is a correlation between the support and the discrepancy of the evidence, as expressed in Eq (15).


$$Sup(m_{i})=1‐d(m_{i})$$ (15)

Therefore, the credible weight of evidence to focal element support can be expressed in Eq (16).


$$C\mathrm{re}(m_{i})=\frac{Sup(m_{i})}{\sum_{i=1}^{n}Sup(m_{i})}$$ (16)

Where, *Cre*(*m*_*i*_)∈[0,1], ∑*Cre*(*m*_*i*_) = 1.

When the credibility weight of evidence to focal element support is high, it shows that the supports of other evidence is to a high degree. The credible weight corresponds to the confidence function in the identification framework, and the product of the credible weight and the confidence function is the reliability of that subsystem. Then the reliability transfer function of the entire system is the product of the subsystem reliability.

#### Fusion algorithm under improved Euclidean distance weighting strategy

The BPA calculation session introduces evidence similarity, evidence difference, and evidence trustworthiness weights to improve the BPA calculation process of the original algorithm. To fully retain the trustworthy conflicts, this part improves the fusion session of the algorithm based on the improved BPA calculation session.

The conflict involvement in fusion directly affects the BPA of the event. Therefore, we construct an improved probability assignment model in terms of the credible weight assignment of conflict information, which uses the product of the credibility of evidence to focal element support and the original probability assignment function. Then, it shows the BPA function of evidence under the new probability assignment model calculated through the BPA calculation session in Eq (17).


$$m_{j}^{\prime }({\vec{A}}_{i})=C\mathrm{re}(m_{i})*m_{j}({\vec{A}}_{i})$$ (17)

By introducing credible conflict, the sum of the fusion results of the relevance of evidence and the fusion results of the credible conflicting evidence makes up a new fusion function. The improved probability assignment function is a fusion calculation of the BPA of the non-conflicting information and the credible conflicting information in the conflict under the new support condition. It means that the improved BPA function is the fusion calculation of the basic probability assignment of the non-conflicting information under the new support condition and the credible conflicting information in the conflict. Thus, the improved probability assignment function is the sum of the BPA function of evidence to a focal element and the support of other evidence to that focal element under the conflict condition, which contains the credible conflict extraction treatment under the new weight for the changed evidence. It shows the new probability distribution function in Eq (18).


$$m_{j}^{\prime \prime }({\vec{A}}_{i})=m_{j}^{\prime }({\vec{A}}_{i})+C\mathrm{re}(m_{i})\sum m_{s}^{\prime }({\vec{A}}_{i})$$ (18)

Where, *s*≠*j*, *i*、*j*、*s*≤*n*. $C\mathrm{re}(m_{i})\sum m_{s}^{\prime }({\vec{A}}_{i})$ denotes the extent to which other evidence agrees with evidence *j* in support of focal element *A*_*i*_.

Normalizing the improved probability assignment to keep the probabilities are in the same mapping environment. Reassigning the weights of conflict and the sum of all evidence probabilities is 1. Under the new conditions, we classify the features of credible conflicts into the category of trustworthy features; the evidence is independent of each other, and the remaining conflicts that are not considered are discardable.

Therefore, the evidence under the new BPA is re-fused, and it shows the fusion rule in Eq (19).


$$E_{1}⊕E_{2}\ldots ⊕E_{n}=\frac{1}{1-K^{\prime }}\sum_{\cap A_{i}=A}\prod_{i\leq j\leq n}m_{j}^{\prime \prime }({\vec{A}}_{i})$$ (19)

Where, $K^{\prime }=\sum_{\cap A_{n}=\phi }\prod_{i\leq j\leq n}m_{j}^{\prime \prime }({\vec{A}}_{i})$, *A*≠∅, indicates that the conflicting factors in the original evidence are involved in the fusion by credible weights.

## Experiment and analysis

### Analysis of improved algorithms in wind turbine operation

Wind power generation technology is mature in renewable energy generation. China has abundant wind energy resources, especially on the southeast coast, Liaotung Peninsula, and northeast. Compared with fossil fuels, the use of clean energy such as wind power can have an effect on reducing carbon dioxide emissions and mitigating global climate change trends. According to a study [51], nearly 80% of power plants in Asia have lost over 30% of their wind energy potential since 1979. Therefore, it takes a wind farm in Jilin province, northeast of China, as an example to analyze the wind turbine operation data, diagnose the fault state, improve system reliability, and increase the efficiency of wind energy utilization.

According to the preliminary analysis, we find that wind speed is one of the key parameters of wind turbine operation; some data showed consistency in the variation pattern; parameters such as pressure and temperature are more sensitive to environmental changes; changes in voltage and current are associated with other parameters, and the overall fluctuations of different wind turbine operations have some similarity. Therefore, we organize and analyze data with a tendency, and select a representative wind turbine in the wind farm to study the parameters such as generator speed, gearbox low-speed bearing temperature, gearbox oil pressure, gearbox inlet oil temperature, and grid current in a certain period. And it does not describe the screening process here. Table 1 shows some of the underlying data sets in the experiment.

**Table 1:** Partial base dataset.

Time	Generator speed	Gearbox low-speed bearing temperature	Gearbox oil pressure	Gearbox inlet oil temperature	Grid current	Wind turbine rotation speed
**1**	**495**	**105**	**1**	**26**	**64**	**46**
**2**	**11025**	**155**	**76**	**19**	**730**	**114**
**3**	**11015**	**257**	**75**	**9**	**626**	**104**
**4**	**10957**	**308**	**74**	**11**	**650**	**104**
**5**	**10974**	**335**	**74**	**15**	**653**	**104**
**6**	**10984**	**352**	**73**	**21**	**626**	**104**
**7**	**11001**	**363**	**72**	**29**	**645**	**104**
**8**	**10999**	**372**	**70**	**39**	**672**	**104**
**9**	**11243**	**379**	**68**	**52**	**818**	**114**
**10**	**12156**	**387**	**68**	**46**	**1618**	**119**
**…**	**…**	**…**	**…**	**…**	**…**	**…**
**…**	**…**	**…**	**…**	**…**	**…**	**…**
**…**	**…**	**…**	**…**	**…**	**…**	**…**
**n**	**11000**	**497**	**43**	**325**	**825**	**100**

When the wind speed is in the steady-state range, the wind turbine speed in the normal operation state of the system is also in the steady-state range. So we analyze the relative change trends of generator speed, gearbox low-speed bearing temperature, gearbox oil pressure, gearbox inlet oil temperature, and grid current during the operation of the wind turbine at a certain time with the wind turbine speed as the base reference parameter. And we find that there is a correlation between the change patterns of some data; the variation trend of different data is different, and the inconsistency of variation shows that there is a conflict between the evidence. Therefore, according to the difference in the changing pattern of data features, we judge whether there is a credible part of the evidence conflict, dig deeply into the consistency information and conflict information in the data, extract credible fault features, analyze the system operation status, and diagnose the system fault.

Let the relative change trends of parameters such as generator speed, gearbox low-speed bearing temperature, gearbox oil pressure, gearbox inlet oil temperature, and grid current are the evidence *E*_*1*_, *E*_*2*_, *E*_*3*_, *E*_*4*_, and *E*_*5*_, respectively. According to the characterization of distinct features, we excerpt valid and representative data periods from the data set, select the basic feature parameters of the evidence in the operation state, and map them into the [0,1] interval to eliminate the influence of data heterogeneity on feature fusion, as shown in Table 2.

**Table 2:** Basic characteristic parameters of evidence.

Evidence	Average	Variance	Root mean square	Cliffness
***E***_***1***_	0.3942	0.1041	2.8362	0.0003
***E***_***2***_	0.2583	0.1080	2.3273	0.0015
***E***_***3***_	0.1043	0.0329	1.1650	0.0114
***E***_***4***_	0.1495	0.0370	1.3562	0.0051
***E***_***5***_	0.1443	0.0557	1.5406	0.0063

In Table 2, we can see that the selected evidence is overall well aggregated. From the variance and root mean square, the dispersion of evidence *E*_*1*_ and *E*_*2*_ is higher than that of *E*_*3*_, *E*_*4*_, and *E*_*5*_; from the cliff factor, the fluctuation of the evidence is roughly a continuous flat change, showing that the data situation is more stable, and it can select the above parameters for the next analysis of the wind turbine.

We divide the mapping of the evidence to system fault support into four types: normal state, implicit fault, explicit fault, and warning. According to the actual occurrence of faults, we identify the points with a more stable change trend in the evidence, define the distribution of the evidence characteristics in the fault characterization, and determine the interval of the fault characterization, as shown in Table 3.

**Table 3:** Fault characterization interval of characteristic parameters.

Evidence	*m(F*_*0*_ *)*	*m(F*_*1*_ *)*	*m(F*_*2*_ *)*	*m(F*_*3*_ *)*
***E***_***1***_	(0,0.0685]	[0.0686,0.2307]	[0.2308,0.6576]	[0.6577,1]
***E***_***2***_	(0,0.0276]	[0.0277,0.1886]	[0.1887,0.5625]	[0.5626,1]
***E***_***3***_	(0,0.0343]	[0.0344,0.0512]	[0.0513,0.1768]	[0.1769,1]
***E***_***4***_	(0,0.0310]	[0.0311,0.1041]	[0.1042,0.3158]	[0.3159,1]
***E***_***5***_	(0,0.0201]	[0.0202,0.0842]	[0.0843,0.2009]	[0.2010,1]

Since 0 in the mapping interval [0,1] contains the cases of the continuous shutdown without starting and shutdown due to fault, we remove element 0 from the normal state *F*_*0*_, which means that it excludes the status data at the moment of normal wind turbine start-up. While processing element 0 in the fault state by adding 1 and classifying it into the warning state *F*_*3*_. The fault interval varies for different systems under different climatic conditions and needs to be determined dynamically based on historical state data.

Organize the data of wind turbine operation, and analyze the fluctuation of data characteristics under different state conditions in the historical data. According to the distribution of the points of the evidence fluctuation interval in different states, such as normal state, hidden fault, explicit fault, and warning, we select a certain moment region with certain credibility and representativeness and calculate the dynamic BPA of each evidence. Depending on the selected interval of the system, it dynamically changes the basic probability distribution. The basic probabilities of selected regions in this paper are calculated and derived, as shown in Table 4.

**Table 4:** BPA values for each evidence.

Evidence	*m(F*_*0*_ *)*	*m(F*_*1*_ *)*	*m(F*_*2*_ *)*	*m(F*_*3*_ *)*
***E***_***1***_	**0.2254**	**0.1765**	**0.4020**	**0.1961**
***E***_***2***_	**0.2549**	**0.3824**	**0.3137**	**0.0490**
***E***_***3***_	**0.4216**	**0.2451**	**0.2255**	**0.1078**
***E***_***4***_	**0.2059**	**0.2549**	**0.4412**	**0.0980**
***E***_***5***_	**0.1765**	**0.3235**	**0.4118**	**0.0882**

From Table 4, it shows that there are different levels of conflicting situations among the evidence, with Evidence *E*_*1*_, *E*_*4*_, and *E*_*5*_ considering the system to have a higher probability of explicit failure, Evidence *E*_*2*_ considering the system to have a higher probability of hidden failure, and Evidence *E*_*3*_ considering the system to have a higher probability of normal state. Exhibit *E*_*5*_ considers that the system also has a higher risk of implicit failure under the high probability of explicit failure.

### Fusion results of the classical algorithm

Based on the typical DST, we fuses the above evidence and it shows the fusion results in Table 5.

**Table 5:** Fusion of traditional DST.

Value	*m(F*_*0*_ *)*	*m(F*_*1*_ *)*	*m(F*_*2*_ *)*	*m(F*_*3*_ *)*
***E***_***1***_ ***、E***_***2***_	0.2204	0.2589	0.4838	0.0369
***E***_***1***_ ***、E***_***2***_ ***、E***_***3***_	0.3449	0.2355	0.4049	0.0147
***E***_***1***_ ***、E***_***2***_ ***、E***_***3***_ ***、E***_***4***_	0.2282	0.1930	0.5742	0.0046
***E***_***1***_***、E***_***2***_***、E***_***3***_***、E***_***4***_***、E***_***5***_	0.1186	0.1838	0.6963	0.0012

From Table 5, we see that after the evidence fused by the original algorithm, the system has a probability of 69.63% of the occurrence of explicit failure, 18.38% of the occurrence of implicit failure, 11.86% of being in a normal state, and a low probability of 0.12% of the occurrence of warning. If evidence *E*_*1*_, *E*_*4*_, and *E*_*5*_ consider the system to have a higher probability of explicit failure, it significantly weakens the support of evidence *E*_*3*_ for the system to be in a normal state and the support of evidence *E*_*2*_ and *E*_*5*_ for the occurrence of implicit failure to some extent. If evidence *E*_*2*_ considers the system to be in a warning state with a lower probability, it weakens the support of evidence *E*_*1*_ for the system to be in a warning state. The trends of the same features strengthen each other and the trends of distinct features weaken each other.

### Fusion results of the algorithm under the improved weighting strategy

Calculate the similarity of the above evidence using a fusion model improved by the weighting method. Then we can get:

$$Si=[\begin{array}{cccc} 0.1689 & 0.1603 & 0.2473 & 0.0475\\ 0.1988 & 0.1432 & 0.1967 & 0.0945\\ 0.1520 & 0.1451 & 0.2972 & 0.0877\\ 0.1390 & 0.1549 & 0.2877 & 0.0804\\ 0.2181 & 0.2064 & 0.1831 & 0.0446\\ 0.1602 & 0.2121 & 0.2557 & 0.0438\\ 0.1451 & 0.2470 & 0.2495 & 0.0428\\ 0.1850 & 0.1767 & 0.2008 & 0.0725\\ 0.1628 & 0.1954 & 0.1978 & 0.0683\\ 0.1340 & 0.2002 & 0.3010 & 0.0656 \end{array}]$$

$$Simz={\left[0.6240,0.6331,0.6819,0.6620,0.6522,0.6718,0.6845,0.6350,0.6242,0.7008\right]}^{T}$$

$$Sim={\left[2.6010,2.6324,2.5445,2.6895,2.6715\right]}^{T}$$

From this, calculate the weight of evidence, as 0.1980, 0.2003, 0.1937, 0.2047, and 0.2033, respectively.

After reassigning the weights, calculate the new BPA values for each piece of evidence: $\left[\begin{array}{cccc} 0.2420 & 0.2323 & 0.3788 & 0.1469\\ 0.2552 & 0.3238 & 0.3395 & 0.0815\\ 0.3296 & 0.2627 & 0.3000 & 0.1076\\ 0.2335 & 0.2672 & 0.3960 & 0.1033\\ 0.3247 & 0.2653 & 0.3377 & 0.0723 \end{array}\right]$.

The fusion results under the new probability are shown in Table 6.

**Table 6:** Fusion results of the improved algorithm under the weighting strategy.

Value	*m(F*_*0*_ *)*	*m(F*_*1*_ *)*	*m(F*_*2*_ *)*	*m(F*_*3*_ *)*
***E’***_***1***_ ***、E’***_***2***_	0.2225	0.2710	0.4633	0.0432
***E’***_***1***_ ***、E’***_***2***_ ***、E’***_***3***_	0.2545	0.2470	0.4824	0.0161
***E’***_***1***_ ***、E’***_***2***_ ***、E’***_***3***_ ***、E’***_***4***_	0.1867	0.2075	0.6005	0.0052
***E’***_***1***_ ***、E’***_***2***_ ***、E’***_***3***_ ***、E’***_***4***_ ***、E’***_***5***_	0.1902	0.1726	0.6360	0.0012

### Fusion results of the algorithm under the improved Euclidean distance weighting strategy

Analyze the above evidence using the algorithm under the improved distance strategy of this paper. Calculate the variance of distinct evidence for event support.


$$d_{ij}=[\begin{array}{cccc} 0.0295 & 0.2059 & 0.0883 & 0.1471\\ 0.1962 & 0.0686 & 0.1765 & 0.0883\\ 0.0195 & 0.0784 & 0.0392 & 0.0981\\ 0.0489 & 0.1470 & 0.0098 & 0.1079\\ 0.1667 & 0.1373 & 0.0882 & 0.0588\\ 0.0490 & 0.1275 & 0.1275 & 0.0490\\ 0.0784 & 0.0589 & 0.0981 & 0.0392\\ 0.2157 & 0.0098 & 0.2157 & 0.0098\\ 0.2451 & 0.0784 & 0.1863 & 0.0196\\ 0.0294 & 0.0686 & 0.0294 & 0.0098 \end{array}]$$

$$d(m_{i})={\left[0.4708,0.5296,0.2352,0.3136,0.4510,0.3530,0.2746,0.4510,0.5294,0.1372\right]}^{T}$$

The normalized variance is:

$d(m_{i})/\sum d(m_{i})={\left[0.1257,0.1414,0.0628,0.0837,0.1204,0.0942,0.0733,0.1204,0.1413,0.0366\right]}^{T}$

Calculate the conflicting credible weights of the evidence, and the credible weights of evidence for event support are 0.1983, 0.1983, 0.1846, 0.2107, and 0.2081, respectively, as shown in Table 7.

**Table 7:** Credibility weight of evidence in support of the event.

Evidence	$d(m_{i}({\vec{A}}_{i}))$	*d*(*m*_*i*_)	*Sup*(*m*_*i*_)	*Cre*(*m*_*i*_)
***E***_***1***_	1.5492	0.2068	0.7932	0.1983
***E***_***2***_	1.5492	0.2068	0.7932	0.1983
***E***_***3***_	1.9610	0.2618	0.7382	0.1846
***E***_***4***_	1.1764	0.1570	0.8430	0.2107
***E***_***5***_	1.2548	0.1675	0.8325	0.2081

Based on the reassigned weights, calculate the new basic probability function values of the evidence, as shown in Table 8.

**Table 8:** The new BPA values for each evidence.

Evidence	*m(F*_*0*_ *)*	*m(F*_*1*_ *)*	*m(F*_*2*_ *)*	*m(F*_*3*_ *)*
***E’***_***1***_	0.2408	0.2323	0.3799	0.1469
***E’***_***2***_	0.2539	0.3240	0.3406	0.0815
***E’***_***3***_	0.3288	0.2627	0.3008	0.1076
***E’***_***4***_	0.2323	0.2673	0.3971	0.1033
***E’***_***5***_	0.3230	0.2659	0.3385	0.0725

From Table 8, we see that it redistributes the probabilities after adopting the trusting attitude to a part of the inter-evidence conflict, the implicit failure rate of evidence *E*_*2*_ decreases, and the explicit failure rate increases; the probability of the normal state of evidence *E*_*3*_ decreases and the probability of explicit failure increases; the probability of the normal state of evidence *E*_*5*_ increases, and the changes of evidence *E*_*1*_ and *E*_*4*_ are smaller. Re-fused them, and it shows the new fusion results in Table 9.

**Table 9:** Fusion results of improved algorithm under Euclidean distance strategy.

Value	*m(F*_*0*_ *)*	*m(F*_*1*_ *)*	*m(F*_*2*_ *)*	*m(F*_*3*_ *)*
***E’’***_***1***_ ***、E’’***_***2***_	0.2201	0.2709	0.4658	0.0431
***E’’***_***1***_ ***、E’’***_***2***_ ***、E’’***_***3***_	0.2501	0.2469	0.4860	0.0161
***E’’***_***1***_ ***、E’’***_***2***_ ***、E’’***_***3***_ ***、E’’***_***4***_	0.1829	0.2069	0.6050	0.0052
***E’’***_***1***_ ***、E’’***_***2***_ ***、E’’***_***3***_ ***、E’’***_***4***_ ***、E’’***_***5***_	0.1850	0.1723	0.6415	0.0012

### Comparative analysis of fusion results under different algorithms

This paper introduces the improved weighting strategy and distance strategy to quantify the correlation and conflict between evidence features in the research process. Compares the fusion results of the original, and improved algorithms, with the actual situation, as shown in Table 10.

**Table 10:** Comparative analysis of fusion results under different algorithms.

Value	*m(F*_*0*_ *)*	*m(F*_*1*_ *)*	*m(F*_*2*_ *)*	*m(F*_*3*_ *)*
***Dempster-Shafer Value***	0.1186	0.1838	0.6963	0.0012
***Weighting strategy value***	0.1902	0.1726	0.6360	0.0012
***Distance strategy value***	0.1850	0.1723	0.6415	0.0012
***Actual target***	0.1695	0.1156	0.6450	0.0699

From Table 10, the evidence after improved algorithm fusion under the weighting and distance strategies, reduces the probability of explicit failure of the system by 5.48% ~6.03% compared with the original algorithm; it reduces the probability of implicit failure by 1.12% ~1.15% and increases the probability of being in a normal state by 6.64% ~7.16%; the probability of early warning is 0.12%, which is consistent with the original algorithm fusion.

**Fig 3:**
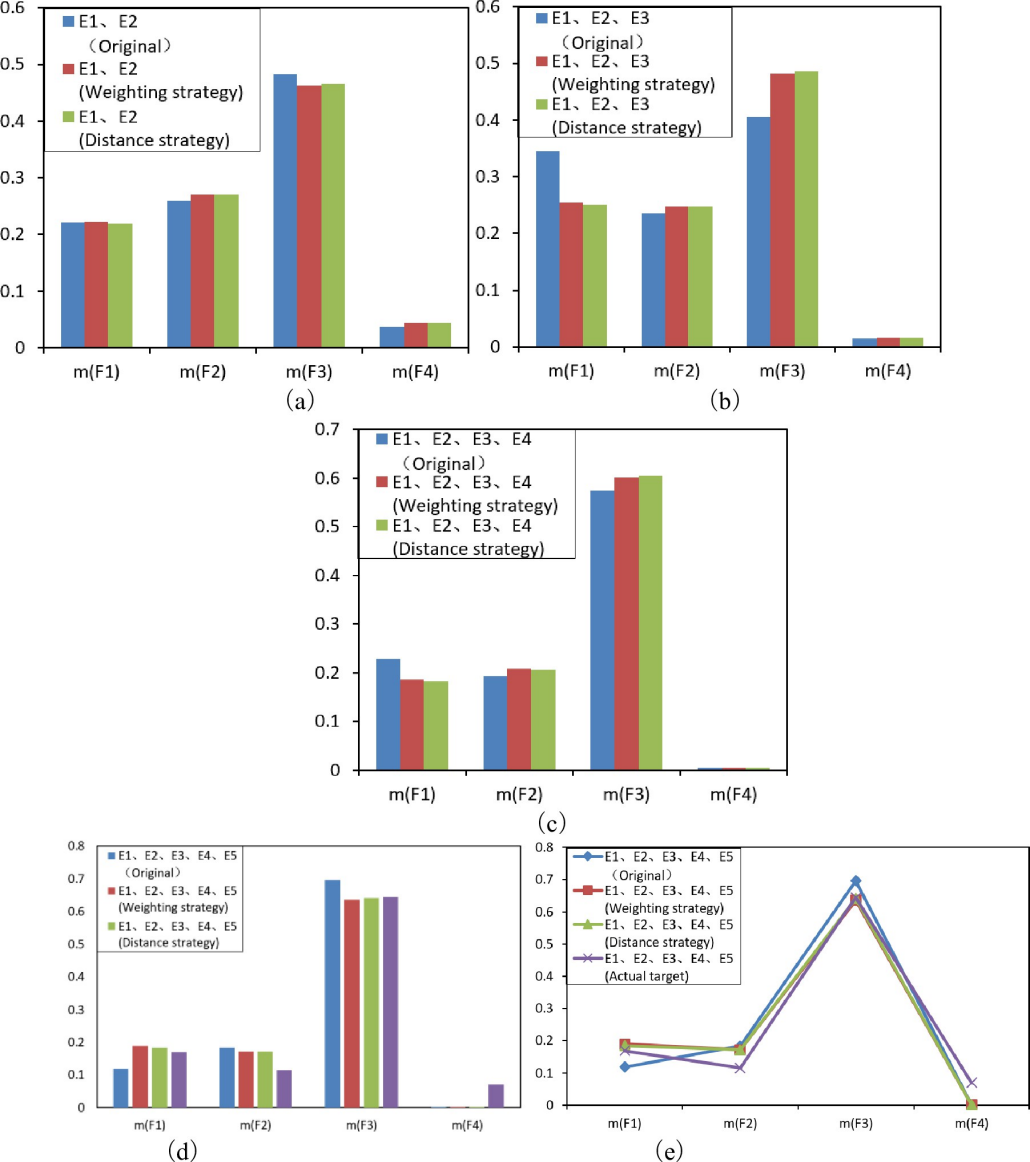
Comparison of evidence fusion results before and after algorithm improvement.

The analysis of the fusion results, as shown in Fig 3, leads to the following conclusions.

1. The changes in the fusion of evidence *E*_*1*_ and *E*_*2*_ before and after the algorithm improved are small, show that the conflicting nature between the two pieces of evidence is small and the conflict participation in the fusion has little impact on the results, as shown in Fig 3(A).
2. In the fusion with evidence *E*_*3*_ and *E*_*4*_, there is a significant change in the judgment that the system is in the *F*_*0*_ state and *F*_*2*_ state. The improved algorithm discards part of the worthless conflicting information in the evidence and absorbs part of the conflicting information in the two states of *F*_*0*_ and *F*_*2*_, leading to a large deviation before and after the improvement, as shown in Fig 3(B) and 3(C).
3. When fused with *E*_*5*_, the probability that the system is in each state shows irregular fluctuations, but overall, the probability that the system is in *F*_*3*_ state has been decreasing, as shown in Fig 3(D).
4. Fig 3(D) and 3(E) demonstrate the gap between the overall trend of fusion change and the actual situation. We can see that the improved fusion algorithm fully considers the conflict factors between the evidence *E*_*2*_ and *E*_*3*_ and *E*_*1*_, *E*_*4*_ and *E*_*5*_ if the evidence *E*_*2*_ and *E*_*3*_ have fully support for the hidden fault and normal states, respectively.

**Fig 4:**
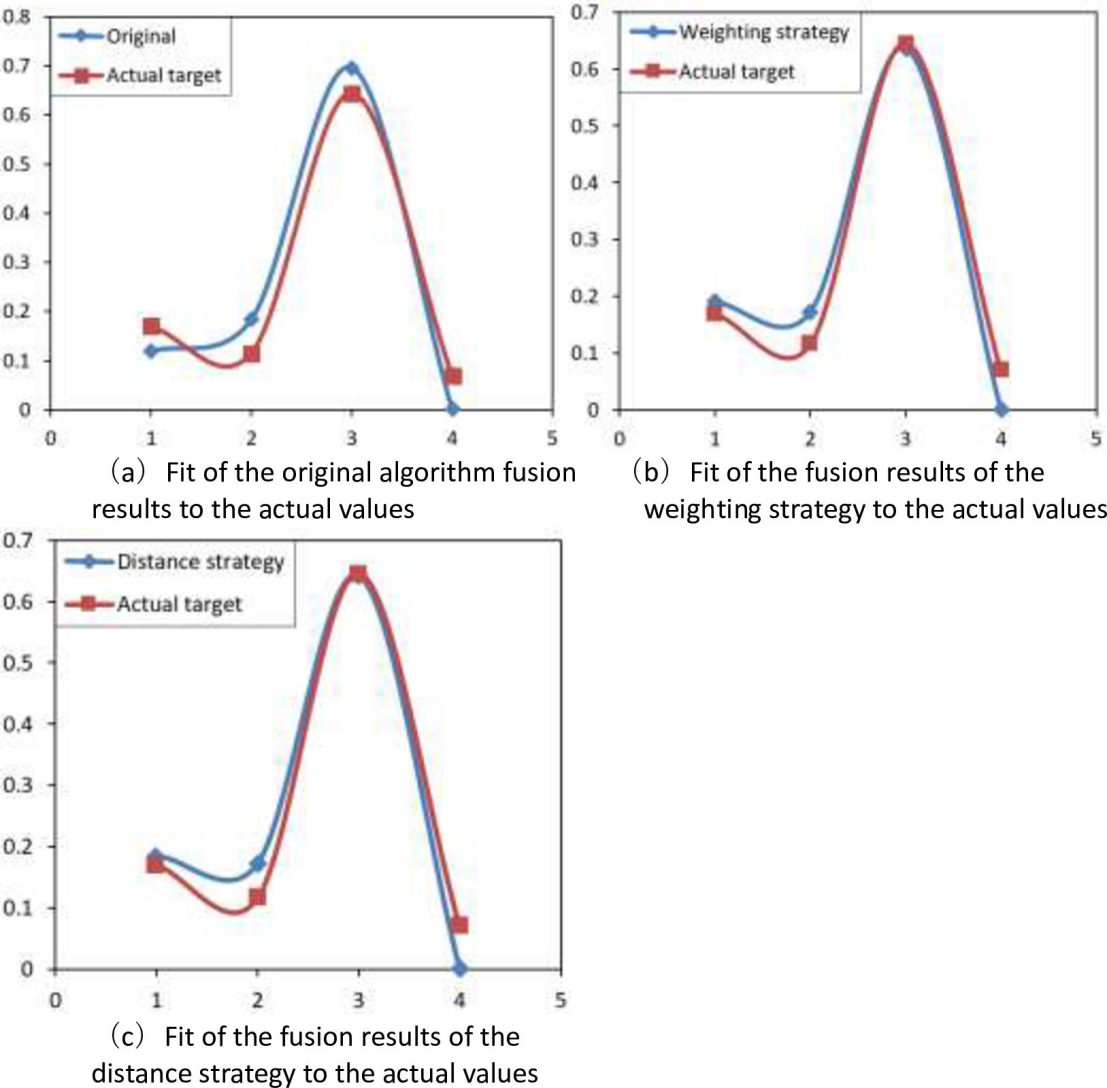
Fit analysis of fusion results.

The stability analysis of the changing trend of the fusion results, as shown in Fig 4, reveals that the original algorithm fusion results fluctuate more with the actual value fitting curve, and the fluctuation of the fusion results with the actual value fitting curve under the weighting strategy and the distance strategy are the same, both improvements have a certain effect, but the improved algorithm under the distance strategy is slightly better than the weighting strategy, and the target value of the fit is better. The improved algorithm under the distance strategy improves the fit with the actual situation by 9.47% compared with the original algorithm, and the improved algorithm under the weighting strategy improves the fit with the actual situation by 8.37%. Overall, the improved algorithm under the distance strategy has better results in diagnosing and predicting system faults and it is more effective in improving energy utilization efficiency.

## Conclusion

In this paper, we propose an improved model of multi-source information fusion under the weighting strategy and distance strategy and check the validity by a case of wind turbine system fault diagnosis in northeastern China. The research results show that the improved algorithm approach under distance strategy has a better adaptability and fits to conflicting information, and quantifies the discrepancy of evidence to event support, credibility, and credible conflict weights considering the fit to reality. The involvement of credible conflicts in the fusion diagnosis solves some uncertainties caused by the loss of credible conflicts and weakens the interference of untrustworthy conflicts on the results.

The proposed algorithm in this paper improves the accuracy of the calculation model, reduces the relevance and uncertainty in the process of using information features, and interprets the practical application significance of the evidence factors after readjusting the basic probability of the evidence. It also improves the scientific and rational system management, enables managers to have a better understand to the system operation status in time. Effectively reducing the system operation and maintenance costs and losses caused by the faults as well as improves the energy utilization efficiency and it has certain advantages in accuracy and timeliness of fault diagnosis.

The method is not only applicable to the wind farm calculations but also to the operational reliability analysis of other energy utilization systems that require comprehensive consideration of multiple factors. Considering the resource utilization efficiency in China, and the complexity and uncertainty of the system operational environment, in the future, we will study the complex system operation reliability in information technology developmentto improve the overall accuracy of the model and realize efficient management of system operation.

## Supporting information

S1 Data (XLSX)

## References

[1] Paggi H, Lara JA, Soriano J. Structures generated in a multiagent system performing information fusion in peer-to-peer resource-constrained networks. Neural Comput & Applic. 2020;32: 16367–16385. doi: 10.1007/s00521-018-3818-1

[2] Shi H, Zhao H, Liu Y, Gao W, Dou S-C. Systematic Analysis of a Military Wearable Device Based on a Multi-Level Fusion Framework: Research Directions. Sensors. 2019;19: 2651. doi: 10.3390/s19122651 31212742 PMC6631929

[3] Fan L. Multiple sensor data fusion algorithm based on fuzzy sets and statistical theory. Zhang W, editor. IFS. 2020;38: 3961–3970. doi: 10.3233/JIFS-179621

[4] Cupek R, Ziebinski A, Drewniak M, Fojcik M. Knowledge integration via the fusion of the data models used in automotive production systems. Enterprise Information Systems. 2019;13: 1094–1119. doi: 10.1080/17517575.2018.1489563

[5] Fu M, Liu J, Zhang H, Lu S. Multisensor Fusion for Magnetic Flux Leakage Defect Characterization Under Information Incompletion. IEEE Trans Ind Electron. 2021;68: 4382–4392. doi: 10.1109/TIE.2020.2984444

[6] Kanmani M, Narasimhan V. An optimal weighted averaging fusion strategy for remotely sensed images. Multidim Syst Sign Process. 2019;30: 1911–1935. doi: 10.1007/s11045-019-00636-9

[7] Mokarram M, Pourghasemi HR, Tiefenbacher JP. Using Dempster–Shafer theory to model earthquake events. Nat Hazards. 2020;103: 1943–1959. doi: 10.1007/s11069-020-04066-w

[8] Denœux T. 40 years of Dempster–Shafer theory. International Journal of Approximate Reasoning. 2016;79: 1–6. doi: 10.1016/j.ijar.2016.07.010

[9] Wu Z, Zhang Q, Cheng L, Tan S. A New Method of Two-stage Planetary Gearbox Fault Detection Based on Multi-Sensor Information Fusion. Applied Sciences. 2019;9: 5443. doi: 10.3390/app9245443

[10] Wen P, Li Y, Chen S, Zhao S. Remaining Useful Life Prediction of IIoT-Enabled Complex Industrial Systems With Hybrid Fusion of Multiple Information Sources. IEEE Internet Things J. 2021;8: 9045–9058. doi: 10.1109/JIOT.2021.3055977

[11] Holst C-A, Lohweg V. Feature fusion to increase the robustness of machine learners in industrial environments. at—Automatisierungstechnik. 2019;67: 853–865. doi: 10.1515/auto-2019-0028

[12] Simjanoska M, Kochev S, Tanevski J, Bogdanova AM, Papa G, Eftimov T. Multi-level information fusion for learning a blood pressure predictive model using sensor data. Information Fusion. 2020;58: 24–39. doi: 10.1016/j.inffus.2019.12.008

[13] Polvara R, Del Duchetto F, Neumann G, Hanheide M. Navigate-and-Seek: A Robotics Framework for People Localization in Agricultural Environments. IEEE Robot Autom Lett. 2021;6: 6577–6584. doi: 10.1109/LRA.2021.3094557

[14] Mokarram M, Khosravi MR. A cloud computing framework for analysis of agricultural big data based on Dempster–Shafer theory. J Supercomput. 2021;77: 2545–2565. doi: 10.1007/s11227-020-03366-z

[15] Hou J, Li Q, Liu Y, Zhang S. An Enhanced Cascading Model for E-Commerce Consumer Credit Default Prediction: Journal of Organizational and End User Computing. 2021;33: 1–18. doi: 10.4018/JOEUC.20211101.oa13

[16] Ai Y-T, Guan J-Y, Fei C-W, Tian J, Zhang F-L. Fusion information entropy method of rolling bearing fault diagnosis based on n-dimensional characteristic parameter distance. Mechanical Systems and Signal Processing. 2017;88: 123–136. doi: 10.1016/j.ymssp.2016.11.019

[17] Qin Y, Xiang S, Chai Y, Chen H. Macroscopic–Microscopic Attention in LSTM Networks Based on Fusion Features for Gear Remaining Life Prediction. IEEE Trans Ind Electron. 2020;67: 10865–10875. doi: 10.1109/TIE.2019.2959492

[18] Zhao X, Jia M, Ding P, Yang C, She D, Liu Z. Intelligent Fault Diagnosis of Multichannel Motor–Rotor System Based on Multimanifold Deep Extreme Learning Machine. IEEE/ASME Trans Mechatron. 2020;25: 2177–2187. doi: 10.1109/TMECH.2020.3004589

[19] Yaghoubi V, Cheng L, Van Paepegem W, Kersemans M. A novel multi-classifier information fusion based on Dempster–Shafer theory: application to vibration-based fault detection. Structural Health Monitoring. 2021; 147592172110071. doi: 10.1177/14759217211007130

[20] Dourado ADP, Lobato FS, Cavalini AA, Steffen V. Fuzzy Reliability-Based Optimization for Engineering System Design. Int J Fuzzy Syst. 2019;21: 1418–1429. doi: 10.1007/s40815-019-00655-5

[21] Xiahou T, Liu Y. Reliability bounds for multi-state systems by fusing multiple sources of imprecise information. IISE Transactions. 2020;52: 1014–1031. doi: 10.1080/24725854.2019.1680910

[22] Suo B, Zhao L, Yan Y. A novel Dempster-Shafer theory-based approach with weighted average for failure mode and effects analysis under uncertainty. Journal of Loss Prevention in the Process Industries. 2020;65: 104145. doi: 10.1016/j.jlp.2020.104145

[23] Jiang W, Wang S, Liu X, Zheng H, Wei B. Evidence conflict measure based on OWA operator in open world. Deng Y, editor. PLoS ONE. 2017;12: e0177828. doi: 10.1371/journal.pone.0177828 28542271 PMC5436833

[24] Brommer C, Jung R, Steinbrener J, Weiss S. MaRS: A Modular and Robust Sensor-Fusion Framework. IEEE Robot Autom Lett. 2021;6: 359–366. doi: 10.1109/LRA.2020.3043195

[25] Xiao F. CaFtR: A Fuzzy Complex Event Processing Method. Int J Fuzzy Syst. 2021 [cited 22 Nov 2021]. doi: 10.1007/s40815-021-01118-6

[26] Wang Y, Liu F, Zhu A. Bearing Fault Diagnosis Based on a Hybrid Classifier Ensemble Approach and the Improved Dempster-Shafer Theory. Sensors. 2019;19: 2097. doi: 10.3390/s19092097 31064125 PMC6540169

[27] Sarabi-Jamab A, Araabi BN. An information-based approach to handle various types of uncertainty in fuzzy bodies of evidence. Calcagnì A, editor. PLoS ONE. 2020;15: e0227495. doi: 10.1371/journal.pone.0227495 31929579 PMC6957153

[28] Ma W, Jiang Y, Luo X. A flexible rule for evidential combination in Dempster–Shafer theory of evidence. Applied Soft Computing. 2019;85: 105512. doi: 10.1016/j.asoc.2019.105512

[29] Lai CS, Tao Y, Xu F, Ng WWY, Jia Y, Yuan H, et al. A robust correlation analysis framework for imbalanced and dichotomous data with uncertainty. Information Sciences. 2019;470: 58–77. doi: 10.1016/j.ins.2018.08.017

[30] Xia F, Tang H, Wang S. Relationships between knowledge bases and their uncertainty measures. Fuzzy Sets and Systems. 2019;376: 73–105. doi: 10.1016/j.fss.2018.11.016

[31] Xiao F. Multi-sensor data fusion based on the belief divergence measure of evidences and the belief entropy. Information Fusion. 2019;46: 23–32. doi: 10.1016/j.inffus.2018.04.003

[32] Zhang L, Ding L, Wu X, Skibniewski MJ. An improved Dempster–Shafer approach to construction safety risk perception. Knowledge-Based Systems. 2017;132: 30–46. doi: 10.1016/j.knosys.2017.06.014

[33] Xiao F. CEQD: A Complex Mass Function to Predict Interference Effects. IEEE Trans Cybern. 2021; 1–13. doi: 10.1109/TCYB.2020.3040770 33400662

[34] Wu X, Duan J, Zhang L, AbouRizk SM. A hybrid information fusion approach to safety risk perception using sensor data under uncertainty. Stoch Environ Res Risk Assess. 2018;32: 105–122. doi: 10.1007/s00477-017-1389-9

[35] Jiang W, Hu W, Xie C. A New Engine Fault Diagnosis Method Based on Multi-Sensor Data Fusion. Applied Sciences. 2017;7: 280. doi: 10.3390/app7030280

[36] Li J, Xie B, Jin Y, Hu Z, Zhou L. Weighted Conflict Evidence Combination Method Based on Hellinger Distance and the Belief Entropy. IEEE Access. 2020;8: 225507–225521. doi: 10.1109/ACCESS.2020.3044605

[37] Tang Y, Zhou D, Xu S, He Z. A Weighted Belief Entropy-Based Uncertainty Measure for Multi-Sensor Data Fusion. Sensors. 2017;17: 928. doi: 10.3390/s17040928 28441736 PMC5426924

[38] Ullah I, Youn J, Han Y-H. Multisensor Data Fusion Based on Modified Belief Entropy in Dempster–Shafer Theory for Smart Environment. IEEE Access. 2021;9: 37813–37822. doi: 10.1109/ACCESS.2021.3063242

[39] Brumancia E, Justin Samuel S, Gladence LM, Rathan K. Hybrid data fusion model for restricted information using Dempster–Shafer and adaptive neuro-fuzzy inference (DSANFI) system. Soft Comput. 2019;23: 2637–2644. doi: 10.1007/s00500-018-03734-1

[40] Xiao F. Generalization of Dempster–Shafer theory: A complex mass function. Appl Intell. 2020;50: 3266–3275. doi: 10.1007/s10489-019-01617-y

[41] Mondéjar-Guerra VM, Muñoz-Salinas R, Marín-Jiménez MJ, Carmona-Poyato A, Medina-Carnicer R. Keypoint descriptor fusion with Dempster–Shafer theory. International Journal of Approximate Reasoning. 2015;60: 57–70. doi: 10.1016/j.ijar.2015.03.001

[42] Elkin C, Kumarasiri R, Rawat DB, Devabhaktuni V. Localization in wireless sensor networks: A Dempster-Shafer evidence theoretical approach. Ad Hoc Networks. 2017;54: 30–41. doi: 10.1016/j.adhoc.2016.09.020

[43] Frittella S, Manoorkar K, Palmigiano A, Tzimoulis A, Wijnberg N. Toward a Dempster-Shafer theory of concepts. International Journal of Approximate Reasoning. 2020;125: 14–25. doi: 10.1016/j.ijar.2020.05.004

[44] Lin Y, Li Y, Yin X, Dou Z. Multisensor Fault Diagnosis Modeling Based on the Evidence Theory. IEEE Trans Rel. 2018;67: 513–521. doi: 10.1109/TR.2018.2800014

[45] Khan MN, Anwar S. Paradox Elimination in Dempster–Shafer Combination Rule with Novel Entropy Function: Application in Decision-Level Multi-Sensor Fusion. Sensors. 2019;19: 4810. doi: 10.3390/s19214810 31694251 PMC6865203

[46] Luo Z, Deng Y. A vector and geometry interpretation of basic probability assignment in Dempster‐Shafer theory. Int J Intell Syst. 2020;35: 944–962. doi: 10.1002/int.22231

[47] Zhao X, Jia Y, Li A, Jiang R, Song Y. Multi-source knowledge fusion: a survey. World Wide Web. 2020;23: 2567–2592. doi: 10.1007/s11280-020-00811-0

[48] Xu W, Yu J. A novel approach to information fusion in multi-source datasets: A granular computing viewpoint. Information Sciences. 2017;378: 410–423. doi: 10.1016/j.ins.2016.04.009

[49] Zhu C, Qin B, Xiao F, Cao Z, Pandey HM. A fuzzy preference-based Dempster-Shafer evidence theory for decision fusion. Information Sciences. 2021;570: 306–322. doi: 10.1016/j.ins.2021.04.059

[50] Li R, Chen Z, Li H, Tang Y. A new distance-based total uncertainty measure in Dempster-Shafer evidence theory. Appl Intell. 2021 [cited 23 Aug 2021]. doi: 10.1007/s10489-021-02378-3

[51] Tian Q, Huang G, Hu K, Niyogi D. Observed and global climate model based changes in wind power potential over the Northern Hemisphere during 1979–2016. Energy. 2019;167: 1224–1235. doi: 10.1016/j.energy.2018.11.027

